# Viscoelastic Property of an LDPE Melt in Triangular- and Trapezoidal-Loop Shear Experiment

**DOI:** 10.3390/polym13223997

**Published:** 2021-11-19

**Authors:** Shuxin Huang

**Affiliations:** 1 Department of Engineering Mechanics, Shanghai Jiao Tong University, Shanghai 200240, China; huangshuxin@sjtu.edu.cn; 2 Key Laboratory of Hydrodynamics of the Ministry of Education, Shanghai Jiao Tong University, Shanghai 200240, China

**Keywords:** LDPE, triangular-loop shear, trapezoidal-loop shear, time-dependent viscoelastic property, Rivlin–Sawyers equation

## Abstract

The time-dependent viscoelastic behaviors of a low-density polyethylene melt (LDPE) in a triangular- and trapezoidal-loop shear experiment reported previously are described here by an integral-type Rivlin–Sawyers (RS) constitutive equation. The linear viscoelasticity of the melt was obtained through a dynamic frequency sweep experiment at a small strain and fitted by a relaxation spectrum. The nonlinear viscoelasticity was characterized by viscosity. All the experimental viscoelastic behaviors of the melt can be divided into two types in terms of the predictions of the RS model: (1) predictable time-dependent viscoelastic behaviors at low shear rates or during short-term shear, and (2) unpredictable shear weakening behavior occurring at the high shear rate of 3–5 s^−1^ during long-term shear with the characteristic time interval of about 40–100 s. The influence of experimental error caused possibly by inhomogeneous samples on the viscoelasticity of the melt was analyzed, and the large relative error in the experiment is about 10–30%.

## 1. Introduction

There are many flow phenomena in the plastic and rubber industry [1], e.g., profile extrusion, film casting, and molding, and the shear viscosity of polymer melts is a fundamental parameter governing these flows since shear viscosity is related to the flow loss or pressure drop in flows. Shear viscosity includes steady and time-dependent for polymer melt, and both are the manifestation of the viscoelastic property of melt. This study aims to extensively understand the transient shear viscoelastic property of a polymer melt, although other viscoelasticity, such as extensional, is also of importance in practice.

The shear viscoelastic properties of variety of commercial or industry-grade polymer melts have been published, e.g., low-density polyethylene (LDPE) [2,3,4,5,6,7,8], linear low-density polyethylene (LLDPE) [9,10], high-density polyethylene (HDPE) [4,5,10,11,12], polypropylene (PP) [13,14,15], polystyrene (PS) [4,16], and polyamide 6 (PA6) [17]. Five types of shear viscoelastic properties are usually included in these publications, which are: (1) linear viscoelastic property, i.e., frequency sweep at small strain, (2) steady shear viscosity, (3) steady first normal stress difference (N_1_), (4) shear stress growth in step rate experiment, and (5) N_1_ growth in step rate experiment. Sometimes, the second steady normal stress difference of polymer melt is reported [7,10], but far less than others because of the difficulty in experimental technique. The published works above—and other similar and unlisted studies not discussed here—raise the understanding of the viscoelastic properties of industrial polymer melts. The applications of the reported experimental viscoelastic properties of polymer melt that are seen in publications are mainly based on two aspects. One aspect is to examine the theoretical model of viscoelastic property of polymer melt [3,5,6,7,8,9,10,11,13,15,16,17,18,19,20,21,22], and the other is to simulate the flow in polymer processing numerically according to the published viscoelastic experimental data [23,24,25,26,27,28].

Constitutive equations are useful in the numerical simulation of polymer processing since constitutive equations provide the stress used in the momentum equation. The accuracy of the theoretical simulation depends on the constitutive equation [25,26]. A large number of constitutive equations have been proposed, and 13 were listed in a recent review [28] on the use of constitutive equations in extrusion cast film processing, in which most are typical and often used in both polymer processing and viscoelastic characterization of the polymer melt, and some can be seen in the references above [3,7,8,9,10,11,13,15,17,19,20,21]. The choice of the constitutive equation in applications depends on the researcher because the researcher usually uses the model that can be used. Due to three factors, i.e., the deficiencies of constitutive equations, the complicated viscoelastic property of polymer melts, and various complex flows in polymer processing, we must modify, remedy, or research the equations in studies [5,6,9,19]. A valid, simple, and accurate constitutive equation is preferred in theoretical simulation in the polymer processing industry.

In 2004, the authors of [29] reported three shear triangular-loop experiments and shear stress growth in a step rate experiment of an LDPE melt at 150 °C, and a modified Huang model [30] was employed to characterize the time-dependent viscoelasticity of the melt. In the following theoretical works [31,32,33,34], some constitutive equations were adopted, modified, or proposed to describe this group of experimental data and to evaluate the capability of these equations. An evident phenomenon is that some equations [31,33] are unable to provide a reasonable explanation for the decreasing stress at a high shear rate during long-term shear. In order to know more about the phenomenon of the LDPE melt, another 16 groups of loop experiments [35] were conducted using both triangular- and trapezoidal-loop shear modes, and five types of flow behaviors were specified according to the forms of the stress–shear rate curves in triangular-loop experiments. Type I flow is a strong shear strengthening behavior and the maximum stress occurs in the ramping-down region of shear rate; Type II is a weak shear strengthening behavior—the maximum stress appears at the maximum shear rate and the stress in the ramping-down region is higher than that in the up region; Type III is that the stress in the down region is almost superposed upon that in the up region at the beginning of ramping-down region; Type IV is that the stress-down curve is lower than the stress-up, and two curves have a cross point, where the shear rate is usually higher than 1 s^−1^ for the melt; Type V is an apparent shear-weakening behavior, not only the stress-down curve is lower than the stress-up, but the shear rate of the cross point is usually lower than 1 s^−1^.

The difference between the step rate experiment and the triangular- or trapezoidal-loop experiment is in the number of variables. Step rate only has a time variable, and loop has two variables of time and shear rate. Therefore, the flow in a loop is slightly complicated. The advantage of the loop experiment is that the unsteady onset in the step rate experiment can be reduced or controlled by the ramping-up process of the shear rate. Greener and Connelly [36] (1986) reported three typical stress–shear rate curves in triangular-loop experiments for a polyacrylamide solution, which did not contain Type I and V flows in [35]. In earlier experimental work of [29], both Type II and III flows were not included. Therefore, the experimental viscoelastic data in [35] can be used to further examine the capability of the constitutive equation, and then promote the understanding of the various time-dependent viscoelastic behaviors of the melt.

The remainder of the paper is organized as follows. Section 2 provides the theoretical analysis of the loop experiment by the Rivlin–Sawyers model [37,38] and the characterization of the viscoelastic properties of the LDPE melt at 150 °C. Section 3 presents the predicted transient viscoelasticities of the melt and the discussions on the shear weakening behavior. Section 4 presents the conclusions of the study.

## 2. Materials and Methods

### 2.1. LDPE

The industry-grade LDPE resin (PE-FSB-23D022/Q200, SINOPEC Shanghai Petrochemical Company Ltd., Shanghai, China) was used in the experiment. The basic characteristics of the plastic are given in Table 1. The mass-average molar mass M_w_, the number-average molar mass M_n_, and M_w_/M_n_ were determined by gel permeation chromatography (GPC).

### 2.2. Setup

The Advance Rheometric Expansion System (ARES, Rheometric Scientific Inc., New Castle, IN, USA) with stainless-steel parallel plates was used to measure the viscoelastic property of the LDPE melt at 150 °C. The diameter of the plate is 25 mm, and the gap size between the parallel plates is about 1.84 mm. The present experimental data only includes the dynamic sweep at small and large strain, and most of the experimental results, i.e., triangular- and trapezoidal-loop data, have been published in [35].

### 2.3. Rivlin–Sawyers (RS) Model

The RS model [37,38] is a type of integral model, and a factorized RS model [38] is simplified here and written as:

(1) $$\sigma =\int_{-\infty }^{t}m\left(t-t^{\prime }\right)\cdot \phi_{1}\left(I_{1},I_{2}\right)\cdot [\delta -C_{t}^{-1}\left(t^{\prime }\right)]dt^{\prime },$$

where *σ* is the extra stress tensor at the present time *t*, m(*t* − *t*′) is the time-dependent memory function, *t*′ is the past time, *ϕ*_1_(*I*_1_, *I*_2_) is a strain-dependent function, *I*_1_ and *I*_2_ are the first invariants of *C*_*t*_^−1^ and *C*_*t*_, respectively, *C*_*t*_ is the Cauchy strain tensor, *C*_*t*_^−1^ is the Finger strain tensor, and δ is the unit tensor. The memory function is given as:

(2) $$m=\sum_{i}\frac{g_{i}}{\lambda_{i}}\cdot e^{\left(-\frac{t-t^{\prime }}{\lambda_{i}}\right)},$$

where *λ_i_* and *g_i_* are the relaxation times and the relaxation modulus coefficients, respectively. The *ϕ*_1_-function is an exponential model, which is written as follows in shear flow [39]:

(3) $$\phi_{1}\left(I_{1},I_{2}\right)=e^{-k\gamma },$$

where *k* is a parameter and *γ* is shear strain. The parameter *k* is obtained by fitting to the experiment in shear flow [31].

**Figure 1:**
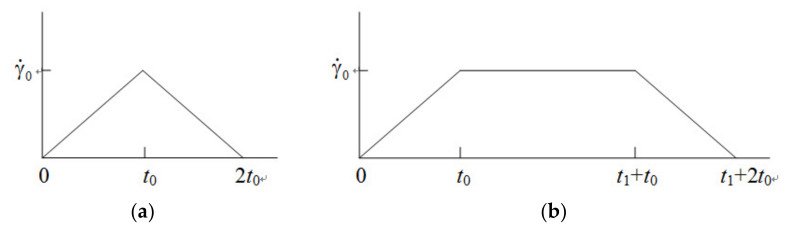
Schematic diagrams of loop experiments, (**a**) triangular-loop, and (**b**) trapezoidal-loop.

The schematic diagrams of loop experiments are shown in Figure 1, where ${\dot{\gamma }}_{0}$ is the imposed maximum shear rate, *t*_0_ is a characteristic time interval of the loop in the ramping-up or -down region of shear rate, and *t*_1_ is a time interval with the constant maximum shear rate. The theoretical analysis of the triangular-loop experiment based on the RS model is given in [31], and therefore, the equations of trapezoidal-loop are presented here.

### 2.4. Shear Strain and Stress in Trapezoidal-Loop

The shear rate in the trapezoidal-loop experiment is,

(4a) $$\dot{\gamma }=a_{0}t,\;\;\;\;\;\;\;\mathrm{for}\;\;\;\;\;\;\;0\;\leq \;t\;\leq \;t_{0},$$

(4b) $$\dot{\gamma }={\dot{\gamma }}_{0},\;\;\;\;\;\;\;\mathrm{for}\;\;\;t_{0}\;\leq \;t\;\leq \;t_{1}+t_{0},$$

(4c) $$\dot{\gamma }=a_{0}(t_{1}+2t_{0}-t),\;\;\;\mathrm{for}\;\;\;t_{1}+t_{0}\;\leq \;t\;\leq \;t_{1}+2t_{0},$$

where *a*_0_ =${\dot{\gamma }}_{0}$/*t*_0_, is the change rate of shear rate in the ramping-up or -down region.

In the ramping-up region (0 ≤ *t* ≤ *t*_0_), the strain history of the trapezoidal loop is,

(5a) $$\gamma (t,t^{\prime })=\frac{1}{2}a_{0}(s^{2}-2ts),\;\mathrm{for}\;t-t^{\prime }\leq t,$$

(5b) $$\gamma (t,t^{\prime })=-\frac{1}{2}a_{0}t^{2},\;\;\;\;\;\;\;\mathrm{for}\;t-t^{\prime }>t,$$

where *s* = *t* − *t*′, is the elapsed time. Equation (5) is the same as that of the triangular loop in [31]. In the steady shear region with the maximum shear rate (*t*_0_ ≤ *t* ≤ *t*_1_ + *t*_0_), the shear strain history is,

(6a) $$\gamma (t,t^{\prime })=-s{\dot{\gamma }}_{0},\;\;\;\;\;\;\;\mathrm{for}\;\;\;t-t^{\prime }\leq t-t_{0},$$

(6b) $$\gamma (t,t^{\prime })=\frac{1}{2}a_{0}(t_{0}^{2}-2t_{0}t+t^{2}-2ts+s^{2}),\;\;\;\;\;\mathrm{for}\;\;\;\;\;t-t_{0}<t-t^{\prime }\leq t,$$

(6c) $$\gamma (t,t^{\prime })=\frac{1}{2}a_{0}(t_{0}^{2}-2tt_{0}),\;\mathrm{for}\;t-t^{\prime }>t,$$

In the down region (*t*_1_ + *t*_0_ ≤ *t* ≤ *t*_1_ + 2*t*_0_), the strain history is

(7a) $$\gamma (t,t^{\prime })=\frac{1}{2}a_{0}s(2t-4t_{0}-s-2t_{1}),\;\mathrm{for}\;t-t^{\prime }\leq t-t_{1}-t_{0},$$

(7b) $$\gamma (t,t^{\prime })=\frac{1}{2}a_{0}(t_{0}^{2}+t_{1}^{2}+t^{2}+2t_{0}t_{1}-2t_{1}t-2t_{0}t-2t_{0}s),\;\;\mathrm{for}\;t-t_{1}-t_{0}\leq t-t^{\prime }<t-t_{0},$$

(7c) $$\gamma (t,t^{\prime })=\frac{1}{2}a_{0}(2t_{0}^{2}+t_{1}^{2}+2t^{2}+s^{2}+2t_{0}t_{1}-2t_{1}t-4t_{0}t-2ts),\;\;\mathrm{for}\;t-t_{0}\leq t-t^{\prime }\leq t,$$

(7d) $$\gamma (t,t^{\prime })=\frac{1}{2}a_{0}(2t_{0}^{2}+t_{1}^{2}+t^{2}+2t_{0}t_{1}-2t_{1}t-4t_{0}t),\;\;\mathrm{for}\;t-t^{\prime }>t.$$

The shear stress in the trapezoidal loop can be calculated using the RS model in terms of the flow history above, which is written as

(8a) $$\tau =\frac{1}{2}a_{0}\sum_{i}\frac{g_{i}}{\lambda_{i}}\int_{\;0}^{\;t}e^{-\frac{s}{\lambda_{i}}}\cdot e^{-\frac{1}{2}ka_{0}(2ts-s^{2})}\cdot (2ts-s^{2})ds+\frac{1}{2}a_{0}t^{2}\cdot e^{-\frac{1}{2}ka_{0}t^{2}}\cdot \sum_{i}g_{i}e^{-\frac{t}{\lambda_{i}}},\mathrm{for}\;0\leq t\leq t_{0},$$

(8b) $$\tau =\sum_{i}\frac{g_{i}}{\lambda_{i}}\int_{\;0}^{\;t-t_{0}}e^{-\frac{s}{\lambda_{i}}}\cdot e^{-k\cdot s{\dot{\gamma }}_{0}}\cdot s{\dot{\gamma }}_{0}\;ds+\frac{1}{2}a_{0}\sum_{i}\frac{g_{i}}{\lambda_{i}}\int_{\;t-t_{0}}^{\;t}e^{-\frac{s}{\lambda_{i}}}\cdot e^{-k\cdot \frac{1}{2}a_{0}(-t_{0}^{2}+2t_{0}t-t^{2}+2ts-s^{2})}\cdot (-t_{0}^{2}+2t_{0}t-t^{2}+2ts-s^{2})\;ds\;+\frac{1}{2}a_{0}(-t_{0}^{2}+2tt_{0})\cdot e^{-k\cdot \frac{1}{2}a_{0}(-t_{0}^{2}+2tt_{0})}\cdot \sum_{i}g_{i}e^{-\frac{t}{\lambda_{i}}},\;\mathrm{for}\;t_{0}\;\leq \;t\;\leq t_{1}+t_{0},$$

(8c) $$\begin{array}{ll} \tau =\frac{1}{2}a_{0}\sum_{i}\frac{g_{i}}{\lambda_{i}}\int_{\;0}^{\;t-t_{0}}e^{-\frac{s}{\lambda_{i}}}\cdot e^{-k\cdot \frac{1}{2}a_{0}s(-2t+4t_{0}+s+2t_{1})}\cdot s(-2t+4t_{0}+s+2t_{1})\;ds\\ \; & \;+\frac{1}{2}a_{0}\sum_{i}\frac{g_{i}}{\lambda_{i}}\int_{\;t-t_{0}}^{\;t}e^{-\frac{s}{\lambda_{i}}}\cdot e^{-k\cdot \frac{1}{2}a_{0}(-t_{0}^{2}-t_{1}^{2}-t^{2}-2t_{0}t_{1}+2t_{1}t+2t_{0}t+2t_{0}s)}\cdot \;(-t_{0}^{2}-t_{1}^{2}-t^{2}-2t_{0}t_{1}+2t_{1}t+2t_{0}t+2t_{0}s)\;ds\\ \;+\frac{1}{2}a_{0}\sum_{i}\frac{g_{i}}{\lambda_{i}}\int_{\;t-t_{0}}^{\;t}e^{-\frac{s}{\lambda_{i}}}\cdot e^{-k\cdot \frac{1}{2}a_{0}(-2t_{0}^{2}-t_{1}^{2}-2t^{2}-s^{2}-2t_{0}t_{1}+2t_{1}t+4t_{0}t+2ts)}\cdot \;(-2t_{0}^{2}-t_{1}^{2}-2t^{2}-s^{2}-2t_{0}t_{1}+2t_{1}t+4t_{0}t+2ts)\;ds\\ \; & \;+\frac{1}{2}a_{0}(-2t_{0}^{2}-t_{1}^{2}-t^{2}-2t_{0}t_{1}+2t_{1}t+4t_{0}t)\cdot e^{-k\cdot \frac{1}{2}a_{0}(-2t_{0}^{2}-t_{1}^{2}-t^{2}-2t_{0}t_{1}+2t_{1}t+4t_{0}t)}\cdot \sum_{i}g_{i}e^{-\frac{t}{\lambda_{i}}} \end{array}\;\mathrm{for}\;\;\;t_{1}+t_{0}\;\leq \;t\;\leq \;t_{1}+2t_{0}.$$

Equation (8a) is also the same as that of the triangular loop in [31]. Equations (8a)–(8c) can be deduced further to obtain an analytical solution. However, it is more convenient to solve Equation (8) numerically, i.e., using the Legendre–Gauss integration method. Therefore, the numerical method was adopted here.

### 2.5. Viscoelastic Characterization of the LDPE Melt

**Figure 2:**
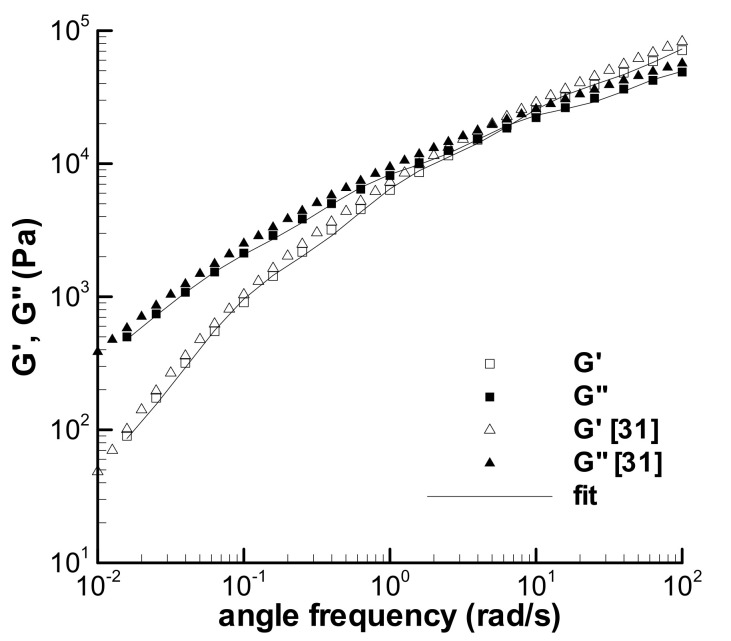
Dynamic frequency sweep experiments of the LDPE (Q200) melt at 150 °C (*γ* = 5%) and its characterization.

As mentioned above in Section 1, a small amount of the time-dependent viscoelasticity of the LDPE melt at 150 °C has been studied in earlier works [29,31]. The difference between the experimental data in [29,31] and those in [35] should be caused by samples, and two batches of the sample sheets molded are used in two experiments. For example, Figure 2 shows the present storage modulus (G′) and loss modulus (G″) obtained in the dynamic frequency sweep experiment at the small strain of 0.05, accompanied by the reported G′ and G″ of the melt [31], and the deviation between two groups of the experimental data could be caused by the samples. Moreover, the experimental data obtained by the samples—cut from one sheet or from one batch of the sheets—also contain errors, which can be seen in the following analysis, making it difficult to analyze the time-dependent viscoelastic behaviors of the LDPE melt.

**Figure 3:**
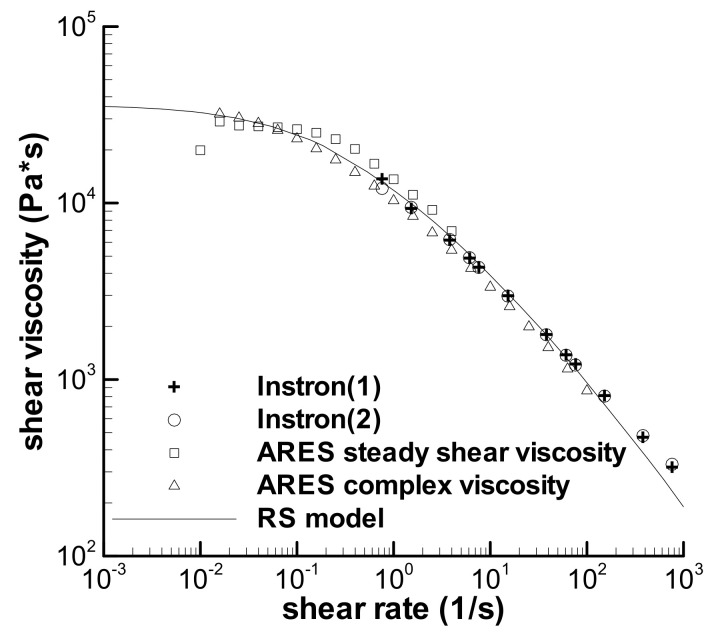
Shear viscosities of the LDPE (Q200) melt at 150 °C and fitting by the RS model.

The characterization of the viscoelastic property of the LDPE melt includes two parts. One is to obtain the relaxation spectrum *λ*_i_ and *g*_i_ in Equation (2) by fitting the frequency sweep data of the melt in Figure 2, and the other is to obtain the parameter *k* in Equation (3) by fitting the viscosities in Figure 3.

The calculated G′ and G″ in Figure 2 describe the present linear viscoelastic property of the melt, which can be used to analyze the loop experiments in [35] because the sample of the G′ and G″ experiment is derived from the same batch of samples used in the loop experiments. The relaxation spectrum fitted is listed in Table 2, together with the previous spectrum [31], and both spectra are different.

Due to the change of linear viscoelasticity, the magnitude of complex viscosity (η*) obtained here is different from that reported [31], which is shown in Figure 3 and denoted by “ARES complex viscosity”. Four groups of the experimental viscosity curves are included in Figure 3, where “Instron(1)”, “Instron(2)”, and “ARES steady shear viscosity” are three groups of the viscosity curves cited from [31], and “ARES complex viscosity” is a set of new data. Thus, the parameter *k* was fitted to the experimental viscosities in Figure 3 and the value of *k* obtained is 0.192. The previous *k* is 0.235 [31]. It is worth noting that many shear-viscosity measurement experiments, not less than 40 groups, have been conducted at different times by an INSTRON-4467 capillary rheometer using a die with the length-to-diameter ratio of 40, and these viscosity curves agree quite well with each other. Therefore, three groups of the viscosity curves reported previously [31] were still used in the present work. The shear-thinning viscosity of the LDPE melt can be described by the RS model, except for some deviation at a shear rate higher than 200 s^−1^.

The influence of experimental errors caused by sample deviation or other unknown factors is usually mixed with the effect of experimental conditions, such as ${\dot{\gamma }}_{0}$, and it is difficult to observe the experimental errors directly among the experimental results. We can discern an error now by assuming a homogeneous effect of the error on an experiment, e.g., a minute defect in a sample could lower the experimental data conducted with the sample, and then, a changing spectrum approach can be adopted here to understand the influence of the error. The homogeneity hypothesis can be seen from the approximate parallel characteristic between the present and the previous G′ and G″ data [31] in Figure 2, thus, the changing spectrum approach can be obtained by modifying the relaxation modulus coefficient with a constant parameter, which can shift the present G′ and G″ approximately to the previous curves. The changing spectrum approach is written as,

(9) $${\lambda^{\prime }}_{i}=\lambda_{i},\;{g^{\prime }}_{i}=f\cdot g_{i}$$

where *f* is the constant. *λ*_i_ and *g*_i_ in Equation (2) will be replaced with *λ*_i_′ and *g*_i_′ as *f* is used. The homogeneous parameter *f* is obtained by fit.

The possible experimental error above is regarded as a sample error in the present work. The deviation between the loop experiments in [35] and those in [29] could be attributed to different batches of the sample sheets, and the deviation only between the loop experiments in [35] could be caused by the inhomogeneous samples—though the samples were cut from a batch of the sheets. The present characterization of the viscoelastic property of the LDPE melt will be used primarily to analyze the experimental errors of the samples in [35] since the same sample batch was adopted in two experiments. The changing spectrum approach employed is similar to that in [40,41,42], and the *f*-parameter in [40,41,42] is a variable.

## 3. Results and Discussion

### 3.1. Validation of the RS Model

In the previous work on the predictions of three triangular-loop experiments [31], Type I flow can be predicted well, however, both Type IV and V cannot. The deviation between the calculation and experiment on Type IV flow is slight. One of the typical results of triangular-loop experiments given by Greener and Connelly [36] in 1986 is also a Type IV flow, which was employed here to testify the validation of the theoretical equation of triangular loop. The maximum shear rate of the Type IV flow—according to Greener and Connelly [36]—is 16 s^−1^ and the time interval *t_0_* is 10 s. The relaxation spectrum of the polyacrylamide solution given by Greener and Connelly is reproduced in Table 3, and the value of the *k*-parameter in the RS model is 0.25, which was obtained by fitting the stress growth tests [36].

**Figure 4:**
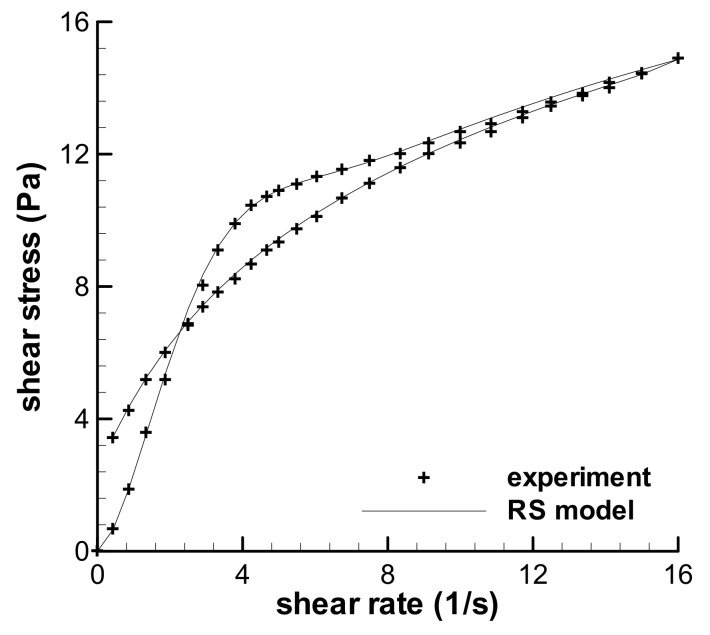
The prediction of the triangular-loop experiment with the maximum shear rate of 16 s^−1^ and the *t*_0_ of 10 s, and the experimental data given by Greener and Connelly [36].

Figure 4 shows the calculated result and the experimental data of the triangular loop experiment of the polyacrylamide solution, and the calculation agrees well with the experiment. This indicates that Type IV flow can be predicted by the RS model and the theoretical analysis on the loop experiment is reliable.

### 3.2. Predictions of the Triangular-Loops

#### 3.2.1. Short-Term Shear with t_0_ = 0.1 s

**Figure 5:**
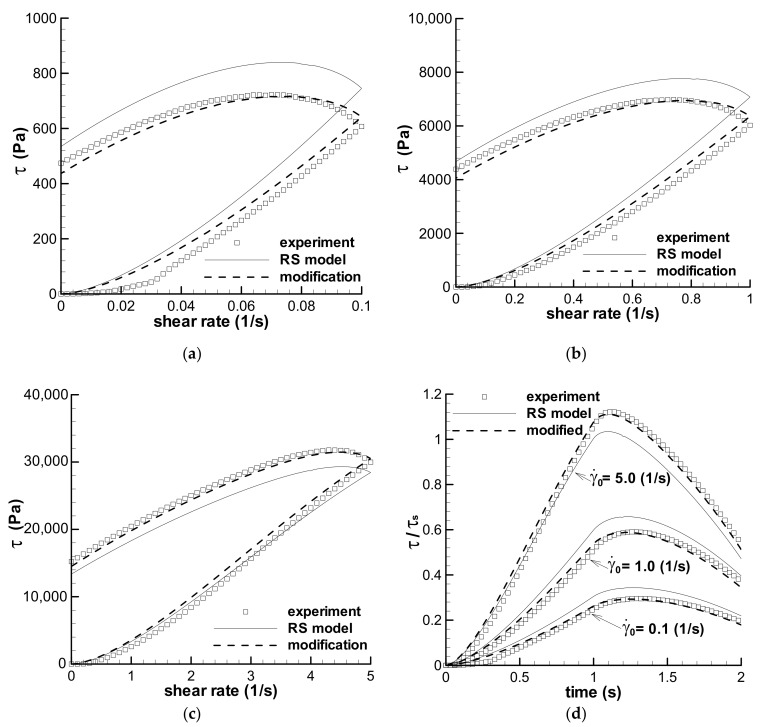
Predictions of the triangular-loop experiments with the same characteristic time *t*_0_ of 1.0 s. The stress–shear rate curves with the maximum shear rates of 0.1 s^−1^ (**a**), 1 s^−1^ (**b**), and 5 s^−1^ (**c**) are given. (**d**) Shows the stress–time curves. Symbols are experiments, solid lines are the calculations by the RS model, and dashed lines are the calculations by the modified spectrum with *f* in Table 4. τ_s_ is the steady shear stress calculated by the RS model at the maximum shear rate.

**Figure 6:**
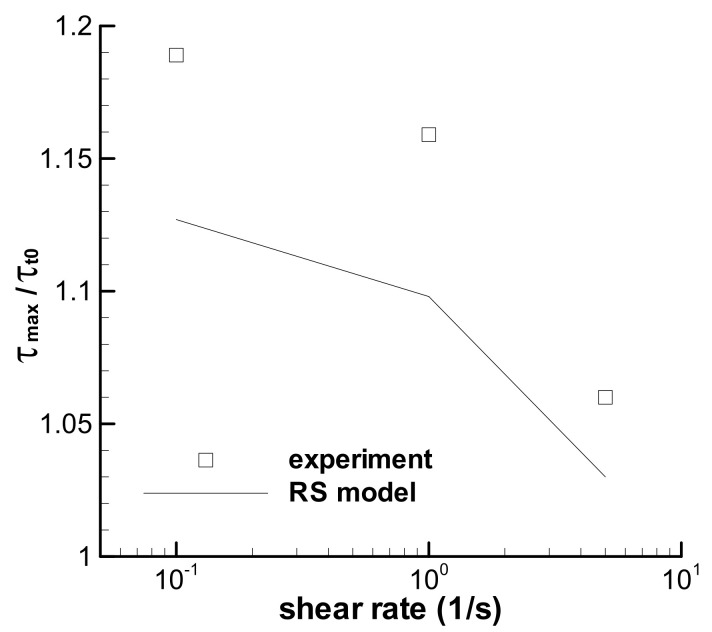
Ratios of the maximum shear stress over the stress at *t*_0_ = 1 s for three triangular loops with *t*_0_ = 1 s. Symbol is the experimental data [35], and line is the calculation.

Figure 5 shows the predictions of the triangular-loop experiments with the same *t*_0_ of 1.0 s for the LDPE(Q200) melt at 150 °C, in which Figure 5a–c shows the stress–shear rate curves, and Figure 5d shows the stress–time curves obtained with three maximum shear rates—0.1 s^−1^, 1 s^−1^, and 5 s^−1^. Evident deviations exist between the calculations of the RS model and the experiments in Figure 5, however, we can still observe three similarities from the calculations and experiments. The first is that both the calculated and the experimental forms of three triangular loops are similar; the second is that three groups of calculations and the corresponding experiments have similar shear–strain strengthening phenomenon, i.e., the maximum stress occurs at a lower shear rate in the ramping-down region, and not at the maximum shear rate; the third is that the ratios of the maximum shear stress (*τ*_max_) over the stress at the maximum shear rate (*τ*_t0_) in three triangular loops with *t*_0_ = 1 s show a similar decreasing trend with increasing shear rate for both the calculated and the experimental data, which are shown in Figure 6.

As stated in Section 2.5, deviations between the calculations and experiments in Figure 5 are regarded as resulting from inhomogeneous samples. Thus, the changing spectrum approach was employed to study the influence of sample deviation, and the calculations with parameter *f* are also shown in Figure 5. We can see that the effect of *f* is significant. The homogeneity hypothesis of the errors should be true, and the changing spectrum method is available to reduce the errors between experiments. The fitted values of *f* are given in Table 4.

According to the stress–shear rate curves in Figure 5, we can see the qualitative contributions of both shear strain and shear rate on the viscoelastic stress. The stress still increases as the shear rate decreases at the beginning of the ramping-down region of shear rate, and the maximum shear stress is not at the maximum shear rate, which indicates the obvious contribution of shear strain on the stress due to the increase in strain. As the shear rate decreases further, the stress decreases with the decreasing shear rate, although shear strain is still increasing. This shows the effect of the shear rate. At the end of the loop, the shear rate is zero and the strain effect is shown again due to the existence of residual stress. Therefore, the LDPE melt is a viscoelastic material because the stress in the triangular loop is controlled simultaneously by both shear strain and shear rate.

Stress growth at a low shear rate in Figure 5 is known here as shear strain strengthening behavior, which is Type I flow and denoted by *τ*_max_/*τ*_t0_ in Figure 6. All three triangular loops show shear strain strengthening behavior under short-term shear, although the maximum shear rate changes from low to high values, and the RS model can describe the phenomenon by excluding the possible experimental errors. Figure 6 also shows that the shear strain strengthening at a low shear rate is more obvious than that at a high shear rate, which can be related to the relative growth of strain, i.e., the ratio of the strain at the maximum stress over the strain at *t*_0_. The ratios of strain growth at the maximum shear rates of 0.1, 1.0, and 5.0 s^−1^ are 1.51, 1.47, and 1.24, respectively, for three triangular loop experiments, and 1.47, 1.39, and 1.19, respectively, for the calculations of the RS model. Both the ratios of strain growth are the decreasing functions of shear rate. The larger the ratio of strain growth is, the higher the shear strain strengthening during short-term shear.

The normalized maximum stresses at the low maximum shear rates, e.g., 0.1 s^−1^ and 1.0 s^−1^, are much lower than 1.0 in Figure 5d, because the shear flows, at low shear rates, do not approach the steady status during short-term shear. This phenomenon can be used to explain the low viscosity at the shear rate of 0.01 s^−1^ in Figure 3, which was obtained by steady shear mode in the ARES rheometer. The low viscosity at 0.01 s^−1^ in Figure 3 could contain error caused by short-term shear.

#### 3.2.2. Medium-Term Shear with *t*_0_ = 10 s

**Figure 7:**
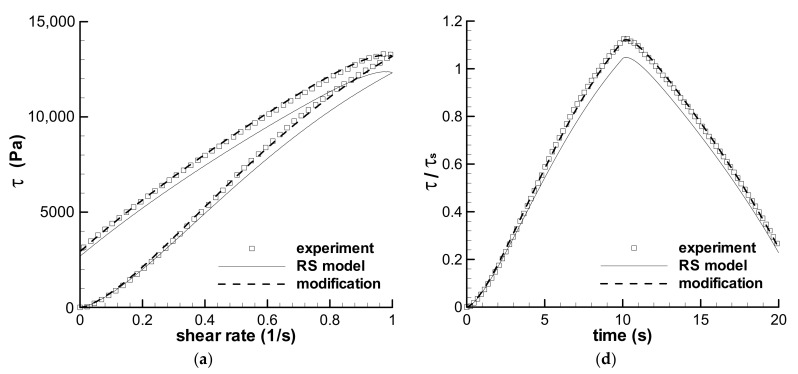

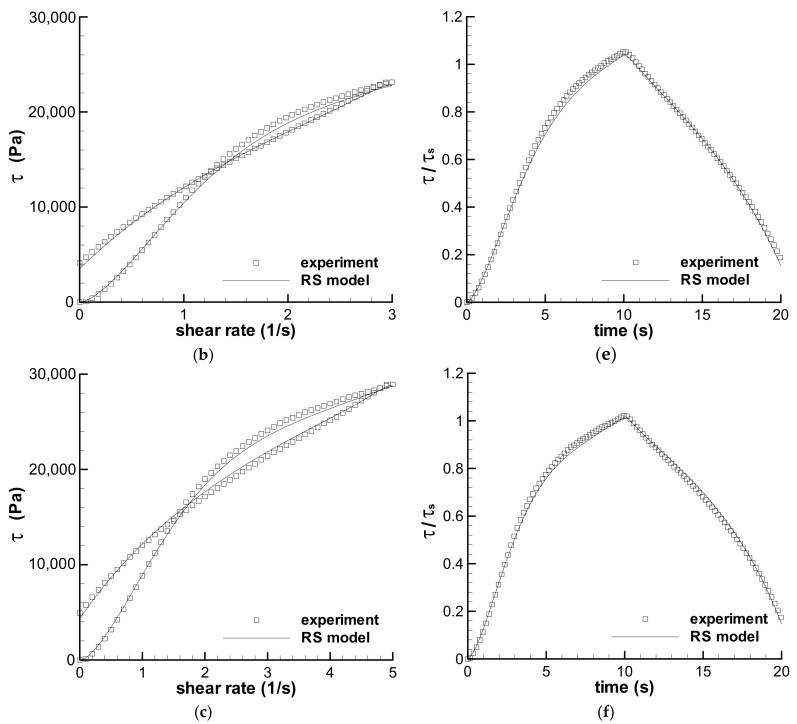
Predictions of the triangular-loop experiments with the same characteristic time *t*_0_ of 10 s. The stress–shear rate curves with the maximum shear rates of 1 s^−1^ (**a**), 3 s^−1^ (**b**), and 5 s^−1^ (**c**) are given. The stress–time curves with maximum shear rates of 1 s^−1^ (**d**), 3 s^−1^ (**e**), and 5 s^−1^ (**f**) are also shown. Symbols are experiments, solid lines are the calculations by the RS model, and dashed lines in (**a**,**d**) are the calculations by the modified spectrum with *f* in Table 4. *τ*_s_ is the steady shear stress calculated by the RS model at the maximum shear rate.

The predictions of the triangular loop experiments with the same *t*_0_ of 10 s are shown in Figure 7, where both the stress-rate curves and the stress–time curves are given simultaneously. Figure 7a shows Type II flow, and Figure 7b,c shows Type IV flow. Two calculations with the maximum shear rates of 3 s^−1^ and 5 s^−1^ are in good agreement with the experimental results, but that with the maximum shear rate of 1 s^−1^ indicates some deviation with the experiment. The influence of inhomogeneous samples could be the reason for the deviation, and therefore, the *f*-parameter was employed to modify the calculation, which shows perfect agreement with the experiment in Figure 7a,d. The fitted *f* for the experiment is listed in Table 4. The time-dependent viscoelastic behaviors of the LDPE melt in the triangular-loop experiments with medium loop time *t*_0_ can be illustrated by the RS model. Moreover, the stress–time curves in Figure 7d–f indicates that the stress at the maximum shear rate has been up to the steady shear status during the ramping-up process of 10 s.

#### 3.2.3. Long-Term Shear with *t*_0_ = 40 s and 100 s

**Figure 8:**
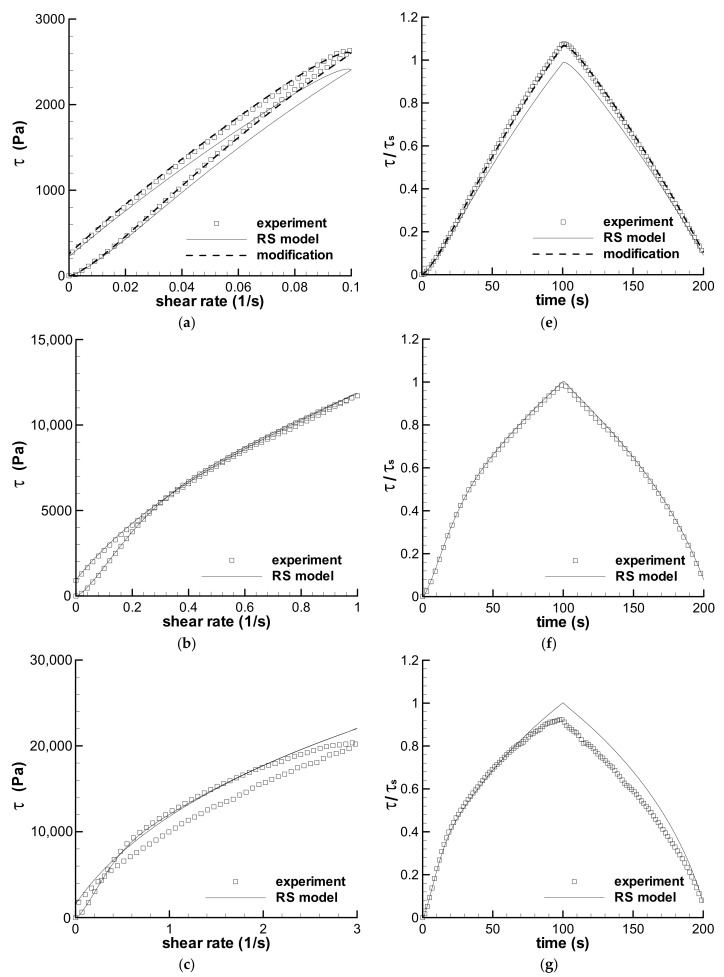

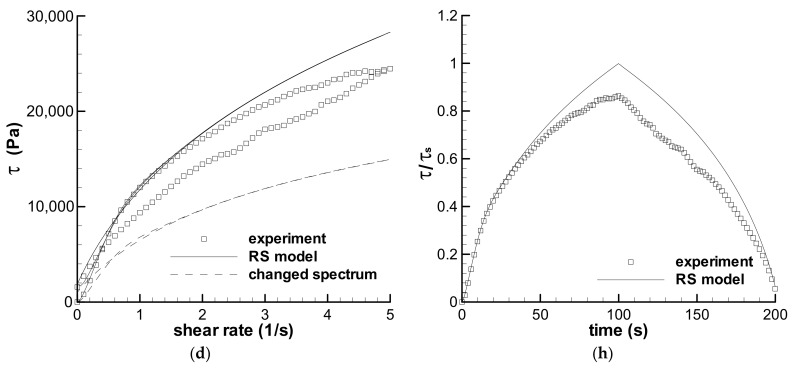
Predictions of the triangular-loop experiments with the same characteristic time *t*_0_ of 100 s. The stress–shear rate curves with maximum shear rates of 0.1 s^−1^ (**a**), 1 s^−1^ (**b**), 3 s^−1^ (**c**), and 5 s^−1^ (**d**) are given. The stress–time curves with the maximum shear rates of 0.1 s^−1^ (**e**), 1 s^−1^ (**f**), 3 s^−1^ (**g**), and 5 s^−1^ (**h**) are also shown. Symbols are experiments, solid lines are the calculations by the RS model, bold dashed lines in (**a**,**e**) are the calculations by the modified spectrum with *f* in Table 4, dashed line in (**d**) is calculated using a changed spectrum in Table 5, and *τ*_s_ is the steady shear stress calculated by the RS model at the maximum shear rate.

The triangular loop experiments with the longest loop-time *t*_0_ of 100 s were predicted by the RS model and are shown in Figure 8. The obvious shear strain strengthening, i.e., the maximum shear stress occurs at a low shear rate, disappears in all the experiments and calculations, however, slight strain strengthening in the ramping-down region of shear rate also appears in the experiment with a maximum shear rate of 0.1 s^−1^ in Figure 8a. The original prediction of the RS model with the spectrum in Table 2 shows deviation from the experiment with a maximum shear rate of 0.1 s^−1^ in Figure 8a, but the modification with parameter *f* shows excellent agreement with the experiment. The *f*-parameter is also listed in Table 4. Figure 8a shows Type II flow, Figure 8b shows Type III flow, and Figure 8c,d show Type V flow. Thus, the predictions in Figure 8 can be divided into two groups. One group is that the calculations of the RS model agree with the experiments with the maximum shear rates of 0.1 s^−1^ and 1.0 s^−1^, i.e., Type II and III flows. The other group is that the calculated stress–shear rate curves do not agree with the experiments with the maximum shear rates of 3 s^−1^ and 5 s^−1^, i.e., Type V flows in Figure 8c,d.

**Figure 9:**
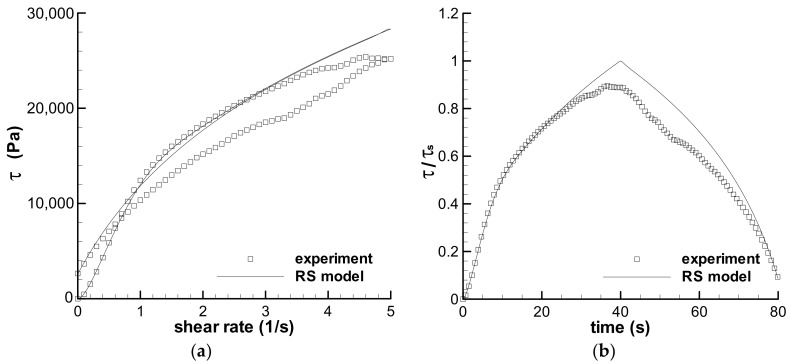
Prediction of the triangular-loop experiment with the characteristic time *t*_0_ of 40 s. The stress–shear rate curve with maximum shear rates of 5 s^−1^ is given in (**a**), and the corresponding stress–time curve is in (**b**). Symbol is experiment, line is the calculation by the RS model, and *τ*_s_ is the steady shear stress calculated by the RS model at the maximum shear rate.

The calculations by the RS model in Figure 8c,d are in good agreement with the experiments at a shear rate lower than about 2 s^−1^ in the ramping-up region of shear rate, and at the following shear rate, the prediction is always larger than the experiment except that at the end of the loop. A feature of the calculated stress–shear rate curves in Figure 8c,d is that the calculated stress curves in the ramping-up region almost overlap those calculated in the ramping-down region at a shear rate higher than that of the cross point of two calculated stress curves. However, the experimental stress curves in Figure 8c,d form an obvious loop at the shear rate higher than that of the cross point of two experimental stress curves, and the experimental stress curves in the ramping-down region are always lower than those in the ramping-up region at the shear rate higher than the cross point. The obvious loop phenomenon in the experiment, in the present paper, is known as shear weakening behavior. A similar loop phenomenon can also be observed in another triangular loop experiment with the maximum shear rate of 5 s^−1^ and a time interval *t*_0_ of 40 s (as shown in Figure 9). The shear weakening behavior cannot be seen in the three stress–shear rate curves of the triangular-loop experiments of Greener and Connelly [36]. Moreover, the RS model cannot describe the shear weakening behavior, i.e., Type V flow, which is influenced by both high shear rate and long-term shear.

Five types of the viscoelastic behaviors specified in the triangular-loop experiments of the LDPE melt [35] can be divided into two types in terms of the calculations by the RS model. One type shows the time-dependent viscoelastic behavior that can be described by the RS model, although some errors appear in the experimental work. Original Type I–IV behaviors [35] belong to the predictable type. The other shows shear-weakening viscoelastic property, i.e., Type V flow, which cannot be described by the RS model.

It is unclear whether the shear weakening viscoelastic property is an intrinsic viscoelastic property of the melt, however, it is worth affirming that part of the viscoelastic data in Type V flow is reasonable according to the comparison of the experiments to the calculations in Figure 8c,d and Figure 9. Therefore, there are two problems with the shear weakening behavior. One is that if the shear weakening is caused by experimental error, the influence of the error should be discerned for obtaining reliable data. The other is that if the shear weakening is a property of the melt, the RS theoretical model is incapable of characterizing the property and should be improved.

### 3.3. Predictions of the Trapezoidal-Loops

Two groups of trapezoidal-loop experiments were also conducted in [35] to understand the time-dependent viscoelastic property of the LDPE melt at 150 °C. As illustrated in Figure 1, *t*_0_ is 1 s in one group, and 100 s in the other. The parameter *t*_1_ is always 200 s for two groups.

#### 3.3.1. Short-Term Startup Shear with *t*_0_ = 1 s

**Figure 10:**
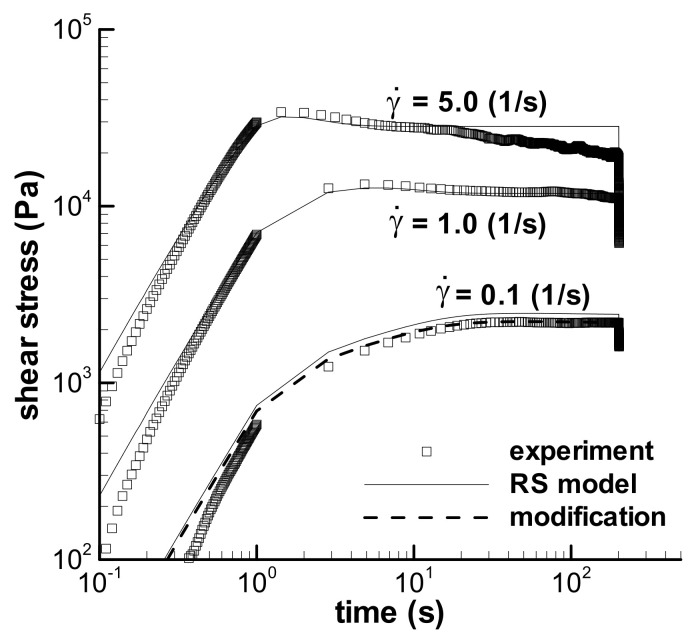
Predictions of the trapezoidal-loop experiments with the maximum shear rates of 0.1 s^−1^, 1 s^−1^ and 5 s^−1^, *t*_0_ = 1 s and *t*_1_ = 200 s. Solid line is the calculation by the RS model, and dashed line is the modification with *f* = 0.875 in Table 4.

Figure 10 shows the predictions of the trapezoidal-loop experiments with maximum shear rates of 0.1 s^−1^, 1.0 s^−1^, and 5.0 s^−1^ and with *t*_0_ = 1 s and *t*_1_ = 200 s. The prediction of the experiment with 0.1 s^−1^ was modified by *f* in Table 4 due to the possible deviation of samples, which is presented in Figure 10 and is in agreement with the experiment. The prediction at the maximum shear rate of 1 s^−1^ also agrees with the experiment. However, the prediction at the maximum shear rate of 5 s^−1^ is in agreement with the experiment only in the time range lower than about 10 s. After 10 s, the calculation approaches constantly, and the experiment drops gradually during long-term shear. The shear weakening behavior appears in the trapezoidal loop experiment with the maximum shear rate of 5 s^−1^, which still cannot be described by the RS model.

**Figure 11:**
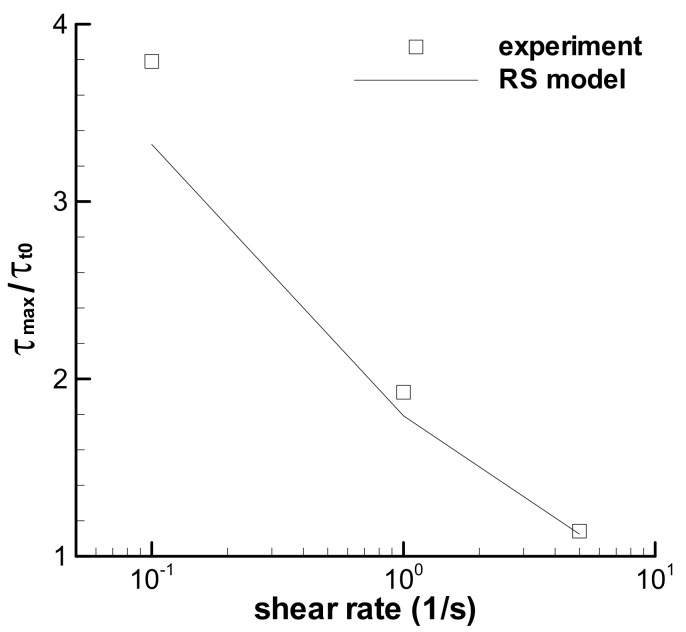
Ratios of the maximum shear stress over the stress at *t*_0_ = 1 s for three trapezoidal loops with *t*_0_ = 1 s. Symbol is the experimental data [35], and line is the calculation. The subscript *t*_0_ denotes the beginning time of the constant shear-rate region, i.e., *t*_0_.

Shear-strain strengthening behavior occurs again in the trapezoidal loop experiments, which behaves as the growth of stress in the constant shear-rate region from the time at *t*_0_ to that at the maximum shear stress in Figure 10. The RS model can describe the strengthening. The ratio of the maximum shear stress *τ*_max_ in the constant shear-rate region over the shear stress at *t*_0_ is employed to quantify the shear strengthening, and the calculated ratios are shown in Figure 11, together with the experimental results [35]. Both groups’ ratios are the decreasing function of the shear rate. Large ratios at low shear rates indicate an obvious contribution of growth of strain on the stress, and elasticity is apparent at a low shear rate for the melt. Moreover, the *τ*_max_/*τ*_t0_ in Figure 11 is much larger than that in Figure 6 at the corresponding shear rate, which indicates the significant effect of the shear rate.

Stress overshoot is usually described in the step rate experiment by the ratio of the maximum shear stress over the steady shear stress during long-term shearing, i.e., *τ*_max_/*τ*_s_ [43,44]. The *τ*_max_/*τ*_s_, i.e., the intensity of overshoot, calculated by the RS model for melt in the trapezoidal loop experiments with *t*_0_ = 1 s, is an increasing function of the shear rate, the same trend as the reported overshoots [43,44]. However, the experimental stress at the maximum shear rate of 5 s^−1^ gradually drops with time, and the experimental overshoot intensity contains part of the shear-weakening effect if we use the stress at a time of 201 s as the steady shear stress to describe the overshoot. Thus, it is challenging to describe the real stress overshoot for the shear-weakening viscoelastic flow at a high shear rate.

#### 3.3.2. Long-Term Startup Shear with *t*_0_ = 100 s

**Figure 12:**
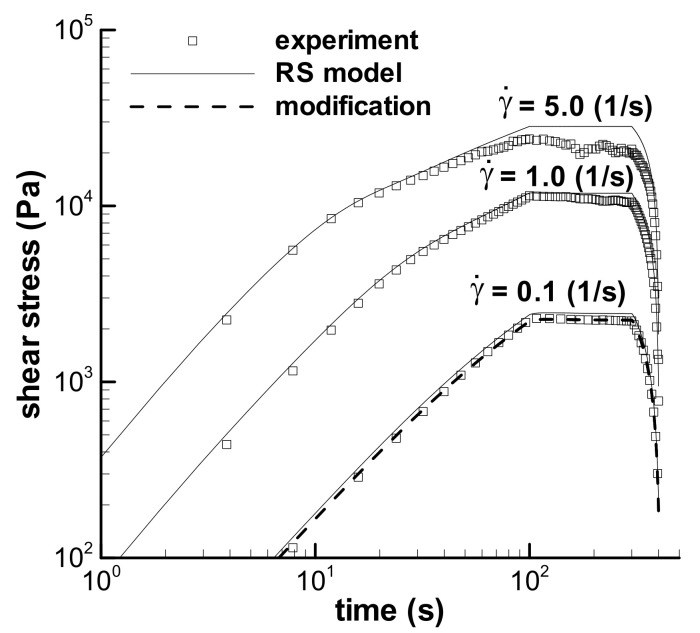
Predictions of the trapezoidal-loop experiments with the maximum shear rates of 0.1 s^−1^, 1 s^−1^ and 5 s^−1^, *t*_0_ = 100 s and *t*_1_ = 200 s. Solid line is the calculation by the RS model, and dashed line is the modification with *f* = 0.899 in Table 4.

The predictions of the trapezoidal-loop experiments with maximum shear rates of 0.1 s^−1^, 1 s^−1^, and 5 s^−1^, *t*_0_ = 100 s and *t*_1_ = 200 s are shown in Figure 12, where the modified calculation of the experiment with 0.1 s^−1^ is also given simultaneously. It is obvious that all three trapezoidal-loop experiments and calculations do not exhibit strain strengthening behavior due to the large *t*_0_. Moreover, both a slight and a severe shear-weakening behavior occur at maximum shear rates of 1 s^−1^ and 5 s^−1^, respectively, during long-term shear. The calculations of the RS model reaffirm the shear-weakening phenomenon of the melt.

According to the experimental and calculated loops, both shear strain strengthening and stress overshoot are time-dependent viscoelastic behaviors. Shear strain strengthening may not be evidently affected by the shear-weakening behavior of viscoelastic fluid due to short-term startup shear, which could be more suitable for illuminating the elastic behavior of fluid; moreover, stress overshoot could contain some shear-weakening effect due to long-term shear sometimes at a shear rate, which complicates stress overshoot.

### 3.4. f Effect

**Figure 13:**
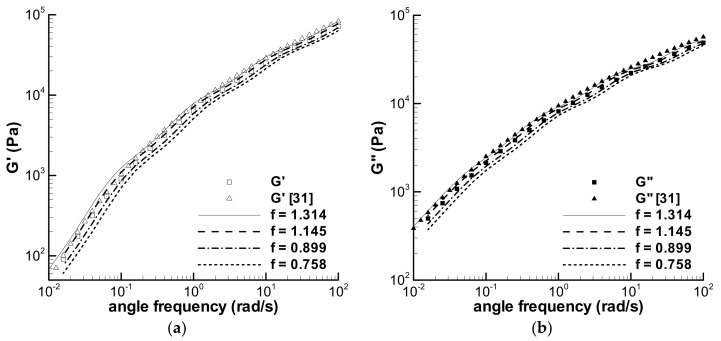
Calculated frequency sweep in small amplitude oscillatory shear mode using some spectra with *f* in Table 4, the present G′ and G″ experimental data (square symbol) and the reported G′ and G″ [31] (triangle symbol). (**a**) G′, and (**b**) G″.

In Section 3.2 and Section 3.3, the influences of possible sample deviations were analyzed using the changing spectrum method, and the modified calculations with *f* in Table 4 by the RS model are in good agreement with the loop experiments. The effects of four *f*-parameters on the original relaxation spectrum in Table 2 are shown in Figure 13, accompanied by the present experimental G′ and G″ in Figure 2 and the previous [31]. The calculations of four changed spectra show an approximate parallel property to the original spectrum. The modified G′ and G″ for the previous frequency sweep experiment with *f* = 1.314 are acceptable although there are some deviations. It is unclear whether the difference between the present G′ and G″ and the previous is caused by the sample, nonetheless, the changing spectrum method is effective.

Eight groups of experiments have been modified with *f* in Table 4, in which the largest *f* is 1.314 for discerning the difference between two frequency sweep experiments. Another *f* varies from 0.758 to 1.145. Therefore, the relative error between the present frequency sweep experiment and the other experiments is about 10–30%, and the definition of relative error is |*f* − 1|/1 × 100. If the error is regarded as sample deviation, the error includes not only the deviation between different batches of the molded sheets but one batch.

### 3.5. Discussion on the Shear Weakening Behavior

It can be assumed that the shear weakening phenomenon of a viscoelastic fluid is the result of a temporal, irreversible change of the structure in the fluid, which can lead to changes in the viscoelastic property during the shearing process. Then, shear weakening behavior cannot be described by the constitutive equation without considering the structure effect on the change in viscoelasticity. In order to understand the structure effect of long-time shearing on the change in linear viscoelasticity, a composite experiment including both the trapezoidal-loop and the dynamic frequency sweep should be conducted. For example, we first conduct the trapezoidal loop experiment with a maximum shear rate of 5 s^−1^, *t*_0_ = 100 s, and *t*_1_ = 200 s, then, simultaneously, we carry out the dynamic frequency sweep at a small strain of 5%. However, the composite cannot be carried out, since the ARES rheometer has a limitation in which the dynamic test must be conducted before the transient or the steady shear test. The startup position of dynamic sweep is fixed for the ARES rheometer, and the position at the end of the loop experiment is unlike that required in the dynamic sweep. No alternative method was found to solve the position problem and the scheme was abandoned.

**Figure 14:**
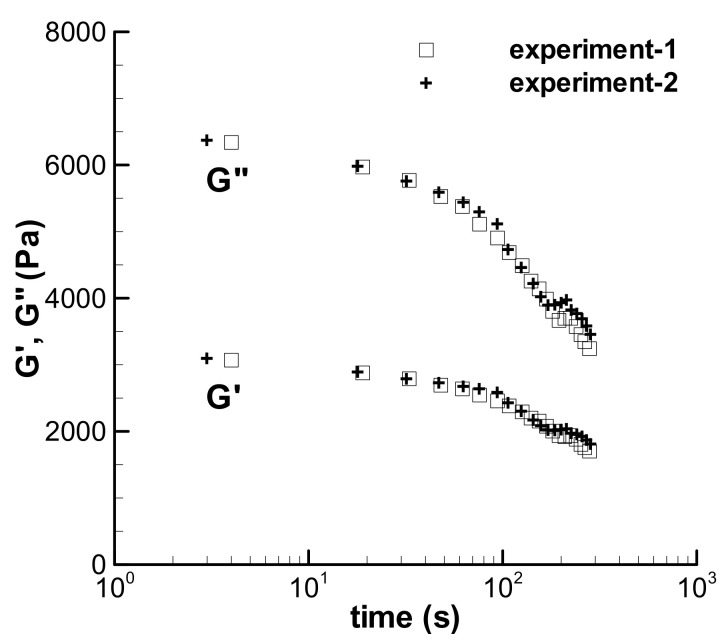
Dynamic time sweeps at large strain, *γ* = 300%, in dynamic shear mode for the LDPE melt at 150 °C (*ω* = 1 rad/s).

**Figure 15:**
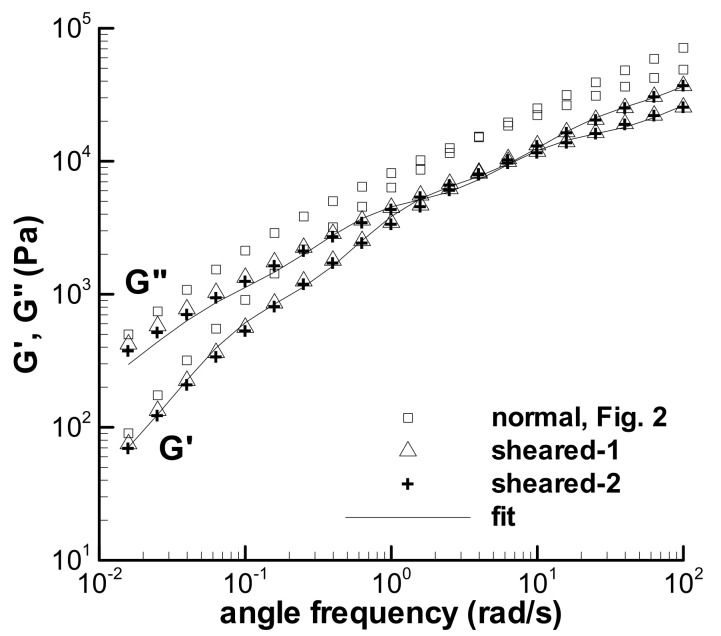
Dynamic frequency sweep experiments at = 5% for the LDPE melt at 150 °C, which are obtained after the time sweeps at the large strain in Figure 14, and the fit on the frequency sweep experiments.

Another approach to qualitatively research the influence of nonlinear large strain shear on the change of linear viscoelasticity for the LDPE(Q200) melt at 150 °C was then adopted, i.e., both the nonlinear and linear shear experiments are accomplished in dynamic mode. A time sweep of 300 s at the large strain of 300% with an angle frequency of 1 rad/s was carried out first, and then, the frequency sweep in small amplitude oscillation shear at a 5% strain was immediately conducted. The experimental G′ and G″ data in two-time sweep experiments at the large strain of 300% are shown in Figure 14, which decrease with time due to the nonlinear shear effect. The G′ and G″ in two small-amplitude oscillation shear experiments obtained upon the cessation of the time sweep of 300 s at the large strain in Figure 15 are reduced by comparing with the normal G′ and G″. The decrease in G′ and G″ in Figure 14 and Figure 15 could be the manifestation of shear weakening phenomenon. The linear viscoelasticity of the melt could be changed by the shear weakening effect in shearing.

To understand the qualitative influence of the altered linear viscoelasticity on the calculation of the loop experiment, a new relaxation spectrum of the LDPE melt was fitted to the G′ and G″ obtained using the shear weakening process in Figure 14 and given in Table 5; the calculated linear viscoelastic property with the new spectrum is shown in Figure 15. Assuming that the nonlinear characteristic of the LDPE melt is not influenced by the shear weakening behavior, the triangular loop experiment with the maximum shear rate of 5 s^−1^ and *t*_0_ of 100 s was calculated again with the spectrum in Table 5 and shown in Figure 8d by a dashed line. The new spectrum evidently reduces the calculated stress of the triangular loop, indicating the qualitative effect of the change of linear viscoelasticity on the description of the shear weakening behavior of the viscoelastic fluid.

The linear viscoelasticity or the relaxation spectrum is unchanged in the characterization of the viscoelastic property of a fluid using the RS model. Thus, the RS model is unsuitable for describing the shear weakening flow behavior of viscoelastic fluid caused possibly by the change of microstructure under large-strain shear. Some structuralized viscoelastic theories [40,42,45,46] may be available in characterizing the shear weakening behavior of the viscoelastic fluid. It is worth noting that the shear weakening in the triangular loop experiment with the maximum shear rate of 5 s^−1^ and *t*_0_ of 100 s in Figure 8d was enveloped by the calculations with both the original spectrum in Table 2 and the new spectrum in Table 5, which indicates that the structure-dependent spectrum approach [40] may be valid in describing the shear weakening behavior.

One crucial issue is whether the shear weakening behavior of the LDPE melt is the manifestation of experimental errors, e.g., edge fracture and loss of material from the gap. Some authors [16,22,47] have emphasized the effect of edge fracture on the shear weakening in the step rate experiment. However, shear weakening may be induced by other factors, such as the shear-structure effect stated above. Previously, shear modification or shear refinement of polythene [48] was reported from an industry viewpoint, and shear modification refers to the reduction in viscoelasticity in shearing, similar to the shear weakening behavior. Rudin [49] (1983) published a review on shear modification, and the authors in [50,51] introduced the possible mechanisms of shear modification. Therefore, the edge fracture effect is still an issue for shear weakening behavior.

## 4. Conclusions

Five types of viscoelastic behaviors specified in the triangular-loop experiments of the LDPE (Q200) melt reported previously [35] were predicted in this study using the Rivlin–Sawyers (RS) model. The viscoelastic properties of the melt were characterized using both the frequency sweep in the small-amplitude oscillation shear experiment and the steady shear viscosities. Type I–IV viscoelastic behaviors can be predicted by the RS model, although some errors appear in the experimental work. Type V flow cannot be predicted, which shows shear-weakening behavior. Most of the calculations agree with the experiments, indicating that both the previous experiments and present theoretical analyses are reasonable.

The trapezoidal-loop experiments of the melt in [35] were also predicted. The shear strain strengthening behavior occurring during the short-term startup shear process in the trapezoidal-loop experiment can be described well by the RS model, which is caused by both the growth of strain and shear rate effect and is similar to that in the triangular-loop experiment. The shear-weakening behavior in the trapezoidal-loop experiment also cannot be described by the model.

Differences between some of the calculations and experiments could be caused by sample deviation, which can be discerned through the changing spectrum approach. The modified calculations with the changing spectrum approach show agreement with the experiments. The large relative error in the experiment is about 10–30%.

The shear weakening behavior of the melt is an issue that can be studied further from two perspectives. One is to study the influence of edge fracture, and the other is to study the effect of changed linear viscoelastic property induced by shear, which could lead to a reasonable description of the shear weakening behavior by theoretical model if the structure effect is real.

## Figures and Tables

**Table 1:** Basic characteristics of the sample.

Material	MFI (g/10 min, 190 °C)	Density (g/cm^3^)	M_w_ (g/mol)	M_n_ (g/mol)	M_w_/M_n_
LDPE-Q200	2	0.922	94,003	9719	9.672

**Table 2:** Relaxation spectra of the LDPE(Q200) melt at 150 °C.

i	*λ*_i_ (s)	Present Work	Ref. [31]
		*g*_i_ (Pa)	*g*_i_ (Pa)
1	10^−4^	2.236 × 10^5^	1.420 × 10^6^
2	10^−3^	1.177 × 10^5^	0
3	10^−2^	6.524 × 10^4^	7.941 × 10^4^
4	10^−1^	2.862 × 10^4^	3.288 × 10^4^
5	10^0^	9.025 × 10^3^	1.051 × 10^4^
6	10^1^	1.620 × 10^3^	1.991 × 10^3^
7	10^2^	6.000 × 10^1^	3.000 × 10^1^
8	10^3^	1.000 × 10^0^	1.000 × 10^0^

**Table 3:** Relaxation spectrum of the polyacrylamide solution given by Greener and Connelly [36].

i	*λ*_i_ (s)	*g*_i_ (Pa)
1	0.0001	25.4
2	0.001	84.5
3	0.01	7.9
4	0.032	7.552
5	0.1	4.8
6	0.32	3.68
7	1	2.06
8	3.2	1.0752
9	10	0.41
10	32	0.1376
11	100	0.0076

**Table 4:** Parameter *f* used in Equation (9) for discerning the obvious experimental errors.

Experiments	*f*
Triangular loop (${\dot{\gamma }}_{0}$ = 0.1 s^−1^, *t*_0_ = 1 s) [35]	0.758
Triangular loop (${\dot{\gamma }}_{0}$ = 1 s^−1^, *t*_0_ = 1 s) [35]	0.819
Triangular loop (${\dot{\gamma }}_{0}$ = 5 s^−1^, *t*_0_ = 1 s) [35]	1.145
Triangular loop (${\dot{\gamma }}_{0}$ = 1 s^−1^, *t*_0_ = 10 s) [35]	1.114
Triangular loop (${\dot{\gamma }}_{0}$ = 0.1 s^−1^, *t*_0_ = 100 s) [35]	1.102
Trapezoidal loop (${\dot{\gamma }}_{0}$ = 0.1 s^−1^, *t*_0_ = 1 s, *t*_1_ = 200 s) [35]	0.875
Trapezoidal loop (${\dot{\gamma }}_{0}$ = 0.1 s^−1^, *t*_0_ = 100 s, *t*_1_ = 200 s) [35]	0.899
Frequency sweep in SAOS [31] ^1^	1.314

^1^ SAOS denotes small amplitude oscillation shear.

**Table 5:** Relaxation spectrum of the LDPE melt obtained after long-term dynamic shear sweep (300 s) at large strain (300%) at 150 °C.

i	*λ*_i_ (s)	*g*_i_ (Pa)
1	10^−4^	2.207 × 10^5^
2	5 × 10^−3^	5.173 × 10^4^
3	5 × 10^−2^	1.409 × 10^4^
4	10^−1^	6.016 × 10^3^
5	10^0^	5.802 × 10^3^
6	1.3 × 10^1^	8.030 × 10^2^
7	10^2^	4.700 × 10^1^
8	10^3^	3.000 × 10^0^

## Data Availability

The data that supports the findings of this study are available within the article.
